# Mating in the cold. Prolonged sperm storage provides opportunities for forced copulation by male bats during winter

**DOI:** 10.3389/fphys.2023.1241470

**Published:** 2023-09-06

**Authors:** Takahiro Sato, Toshie Sugiyama, Tsuneo Sekijima

**Affiliations:** ^1^ Graduate School of Technology, Industrial, and Social Sciences, Tokushima University, Tokushima, Japan; ^2^ Faculty of Agriculture, Niigata University, Niigata, Japan

**Keywords:** reproductive delay, heterothermy, sperm competition, sexual coercion, Chiroptera

## Abstract

In a wide range of heterothermic mammals, hibernation interrupts the reproductive cycle by forcing reproductive delays. In hibernating bats with delayed fertilization, an opportunity for sperm competition is enhanced by extending a time-window between copulations and fertilization. In order to achieve greater fertilization success, males are expected to show adaptations for sperm competition by increasing their opportunities for mating over an extended period. We aimed to clarify the physiological and behavioral characteristics of male bats experiencing increased risks of sperm competition. We investigated the characteristics of the reproductive cycle of the little horseshoe bat (*Rhinolophus cornutus*), and examined whether males retain reproductive physiology related to sexual behavior, and attempt to copulate with females even during the hibernation period. Field observations and histological examinations of the reproductive cycle confirmed that females, having mated in the autumn, store spermatozoa in the uterus during hibernation and give birth in the early summer to just one offspring per year, thus males face a low certainty of successful fertilization. Although their testes regressed rapidly and their testosterone levels were lower during winter than in autumn, males stored motile spermatozoa in their cauda epididymides from autumn throughout the winter. During hibernation, we found that males occasionally aroused from torpor and attempted to mate forcibly with torpid females. Forced copulations appear to increase a male’s chances of obtaining a mate while avoiding pre-copulatory female choice. Epididymal sperm storage could be advantageous for males in allowing them to extend their potential mating period even though their testes have regressed. We also found that some hibernating nulliparous females were ready for fertilization in spring after hibernation, whereas few parous females appeared in the same roost. In contrast to males, forced copulations would be maladaptive for females because they cannot opt for higher-quality males while in torpor. Females that have experienced sexual coercion when young may subsequently avoid hibernacula where adult males are present.

## 1 Introduction

A wide range of heterothermic mammals conserve energy so as to survive the season of low ambient temperature and scarce prey availability by entering deep, multi-day torpor (i.e., hibernation) (Geiser, 2013). Hibernation is characterized by a regulated reduction in metabolic rate and body temperature resulting in a depression of energy expenditure (Geiser, 2004; Ruf and Geiser, 2015). The physiological state of hibernation slows down many endocrine activities leading to the suppression of circulating levels of reproductive hormones and the suppression of the functions of the reproductive organs (Racey and Swift, 1981; Barnes et al., 1986; Gagnon et al., 2020). Therefore, it has been presumed that hibernation and reproduction are mutually incompatible processes, particularly among rodents (Wimsatt, 1969).

Increasing knowledge of the relationship between thermoregulation and reproductive status has recently shown that hibernation and reproduction can be compatible in some mammal species (McAllan and Geiser, 2014; Geiser, 2021). Among marsupials and bats, females often enter torpor during pregnancy and lactation (Geiser et al., 2005; Körtner et al., 2008; Dzal and Brigham, 2013). Although bouts of torpor during the reproductive season are mostly shallower and shorter than during winter, pregnant hoary bats (*Lasiurus cinereus*) enter multiday torpor, which meets the common definition of hibernation (Willis et al., 2006). Entering torpor temporarily during the reproductive period allows insectivorous species, such as bats, to avoid starvation caused by prey scarcity during extreme weather conditions (Stawski et al., 2014). Reproductive cycles overlap hibernation in Tasmanian echidna (*Tachyglossus aculeatus*) and temperate zone bat species (Crichton, 2000; McAllan and Geiser, 2014). In Tasmanian echidna, males have enlarged testes and are likely to mate with females during hibernation (Morrow and Nicol, 2009; Morrow et al., 2016). Interesting phenomena co-occurring reproduction and hibernation are reproductive delays in bats (Racey and Entwistle, 2000). Among bats, reproductive events are interrupted by prolonged winter torpor, thereby extending the period, either between copulation and fertilization (delayed fertilization) or between fertilization and implantation (delayed implantation) (Oxberry, 1979; Sandell, 1990). The physiological mechanisms that allow bats to maintain reproductive functions during delays have been discussed intensively in terms of the anatomical features of their reproductive organs and the involvement of sex hormones (e.g., androgen for female sperm storage) (Racey and Entwistle, 2000; Ocampo-González et al., 2021). In contrast, their reproductive strategies or tactics during hibernation, which are facilitated by reproductive delays, remain to be discussed.

A unique feature of bats exhibiting reproductive delays is that their opportunities for post-copulatory sexual selection are enhanced (Orr and Zuk, 2014; Orr and Brennan, 2015). In those bat species with delayed fertilization, mating occurs during late-summer and autumn before hibernation, but ovulation and fertilization occur in spring after hibernation (Racey, 1979). During the delay, females store sperm for several months within their reproductive tract (Racey, 1973; Wang et al., 2008). As bats typically bear only one or two offspring per litter (Barclay and Harder, 2003), in those species in which females mate multiple-times, males face reduced certainty of fertilization success. Thus, intense male-male competition is facilitated in the form of sperm competition. Male reproductive traits are under strong selection pressure (Orr and Zuk, 2014). For example, species with delayed fertilization have larger testes than those without delays, indicating that males invest more resources in sperm production (Orr and Zuk, 2013). Bat species in which there is a greater risk of sperm competition, including species with delayed fertilization, have elaborate penile morphology (e.g., the presence of penile spines) and sperm features related to competitive ability (e.g., sperm length and mitochondria quantity) (Hosken, 1997; Fasel et al., 2019). Since delayed fertilization extends the time-window from copulation until fertilization, the hibernating season potentially provides an extended period for mating. In order to achieve greater fertilization success, males are expected to exhibit characteristics that allow them to obtain more mating opportunities. Hibernation certainly inhibits many reproductive processes. However, even under such constraints, physiological and behavioral traits that maximize reproductive success could evolve through sexual selection, particularly in bats with intense sperm competition. If males are able to retain reproductive functions related to sexual behavior even during hibernation, they may be able to copulate over an extended period.

While previous research has often focused on morphological traits (e.g., testes size) as an index of sperm competition, little effort has been made to investigate physiological and behavioral characteristics integrally in the context of male adaptations to sperm competition during hibernation. As a model species to test our predictions, we focused on the cave-dwelling little horseshoe bat (*Rhinolophus cornutus*), which is widely distributed in the Japanese archipelago (Sano and Armstrong, 2009). In this species females give birth to a single offspring in early summer (mainly in late June) (Sano and Armstrong, 2009). Although it was not known whether little horseshoe bat exhibits delayed fertilization or not, sperm competition could be enhanced if females store sperm during hibernation. The anatomical study of male reproductive organs suggested that sperm may be retained within the epididymides during winter (Kurohmaru et al., 2002). Since 2011, we have also monitored the reproductive cycle of a wild population of little horseshoe bat, and we observed torpid males with enlarged external gonads during winter. We expected that males were able to store sperm and attempt to copulate during hibernation.

In this study our first aim was to describe the annual reproductive cycle of the little horseshoe bat, and to investigate whether females store sperm during hibernation, which would be a characteristic of delayed fertilization. Our second aim was to clarify whether or not males retain reproductive conditions enabling them to mate during winter (as well as in autumn). We also tested the prediction that copulation occurs during hibernation. Furthermore, histologically, we determined the reproductive conditions of wintering females to examine whether females were fertilizable in spring, and thus potential mates for males.

## 2 Materials and methods

### 2.1 Study site

Our field work was conducted in Osawa cave (37° 40′N, 139° 06′E, 216 m above sea level) located in Gosen City, Niigata Prefecture, Japan. The ambient temperature in the cave was stable throughout the year (mean ± SD = 9.9°C ± 0.4°C, recorded at 1-h intervals from June 2011 to May 2012 using a temperature data logger). The total number of little horseshoe bats in the cave peaked during summer and winter (700–900 individuals), and was lowest (<10–200 individuals) in late autumn from September to November. Although no other roosts have been found in the vicinity of Osawa cave, it seems that most of bats in the colony disperse to other roosts during autumn. We also collected specimens for experiments assessing male reproductive physiology (section 2.5 in detail) from an abandoned tunnel (37° 08′N, 138° 38′E, 169 m above sea level) located in Tokamachi City, Niigata.

### 2.2 Ethics statement

All capture, handling and sampling procedures were approved by the Kanto Regional Environmental Office in Saitama, Ministry of the Environment (Permit Number: #1311211, #1405126, #1505111, #16052311 and #1705245), Niigata Prefecture (Permit Number: #148, #208 and #282) and Gosen City (Permit Number: #37). All of the animal experiments were carried out in compliance with procedures reviewed by the Institutional Animal Care and Use Committee and approved by the President of Niigata University (Permit Number: Niigata Univ. Res. 356-2, 356-3, and 356-4).

### 2.3 Determination of annual reproductive cycle

To determine the annual reproductive cycle of the little horseshoe bat in our study site, preliminary investigations of the hibernation period were conducted from August 2011 to July 2012. We used a Thermo Gear G120 (Nippon Avionics, Kanagawa, Japan) thermal imaging camera for monitoring the surface body temperatures of bats. When possible, we placed the camera on the cave floor 4–7 m away from a group of bats containing >50% of the total number of individuals in the cave, and observed bats for 5–7 days each month. The camera was programmed to take a thermal image (±0.1°C resolution) every 10 min. Thermo Gear G120 has a horizontal field of view of 32° and a vertical field of view of 24° with 1.78 mrad spatial resolution (17.8 mm/pixel at 10 m from a target). Thermal images were confirmed and analyzed using InfRec Analyzer NS9500 Lite software (Nippon Avionics). The camera was powered by a 12V100Ah sealed battery.

The sensitivity of a thermal imaging camera decreases with distance due to absorption of radiant energy from an object, particularly in an environment with high relative humidity such as inside a cave. To detect whether bats being observed became euthermic in thermal images, we determined a temperature threshold using the following procedure. First, we captured bats during active (September 2011, 10 males and 10 females) and inactive periods (January 2012, 14 males and 5 females). When capturing bats, we chose individuals making noticeable movements during the active period, and individuals not moving (i.e., torpid) during the inactive period. The rectal temperature of captured bats was measured using a thermometer connected to thermistor probe (active: mean ± SD = 30.7°C ± 2.0°C, range = 26.9°C–33.4°C; torpid: mean ± SD = 11.7°C ± 1.5°C, range = 10.2°C–15.9°C). Then, for each individual, the surface body temperature was immediately measured in the cave at distances of four and 7 m. Second, we compared the surface body temperature of active and torpid bats to test whether they are distinguishable from hibernating bats. The mean surface body temperature of active bats was significantly higher than that of torpid bats (Welch two sample *t*-test at 4 m: *t* = 29.67, *df* = 19.33, *p* < 0.0001, and at 7 m: *t* = 28.44, *df* = 19.94, *p* < 0.0001). The surface temperature range of active bats (14.9°C–21.1°C at 4 m, 13.1°C–18.2°C at 7 m) did not overlap that of torpid bats (7.6°C–8.2°C at 4 m, 7.6°C–8.3°C at 7 m). Therefore, we considered bats to be “aroused” once their surface body temperatures exceeded the minimum value of active bats (14.9°C at 4 m, 13.1°C at 7 m). For our purposes we have defined hibernation as bats in deep multiday torpor without exceeding the temperature threshold for more than 24 h. We recorded the presence or absence of hibernating bats in the observed group in each month. Based on our monthly monitoring, torpid bats fulfilling our definition of hibernation were observed exclusively from December to March, allowing us to classify the little horseshoe bat’s annual life cycle as active from April to November and in hibernation from December to March (Supplementary Figure S1).

The reproduction of the bats was studied from June 2011 to March 2017. We captured bats once each month in Osawa cave using a hand-net or a mist-net (2.6 m heigh, 6.0 m wide, 35 mm mesh, Tokyotobari, Tokyo, Japan). Before capture, we took images of bats in the cave using a digital camera (DSC–RX100M2, Sony Corporation, Tokyo, Japan) to record their roosting positions, and to count the total number of individuals. When hand-netting bats, we minimized the duration of our visits to the cave to less than 20 min to avoid excessive disturbance. Bats were handled and processed at the cave entrance. Little horseshoe bats form their nursery colonies from late June to early August. To minimize disturbance to the colony during this period, a single mist-net was positioned near the cave entrance just before sunset, and retrieved 1 h after sunset.

The sex, age class, and reproductive condition of captured bats were identified. Individuals were assigned to three age classes, juveniles, subadults, or adults, based on wing epiphyseal fusion in the finger bones and their degree of sexual maturity. Juveniles are defined as young of the year with incomplete epiphyseal fusion of their finger bones. Subadults have complete ossification of their finger joints but are still sexually immature. Adults have fully developed finger bones and are sexually mature. Identification of sexual maturity was based on observations of the external genitalia of captured individuals. In rhinolophid bats, females with experience of lactation have a pair of pubic nipples (to which the pups cling) at the upper part of the vulva. Thus, we regarded the presence of pubic nipples as a sign of sexual maturity in females (hereafter referred to as “parous”). Females without pubic nipples were identified as nulliparous. We also classified the reproductive condition of subadult and adult females as pregnant, lactating, or indistinguishable, by palpating the abdomen or confirming hair loss and enlargement of nipples and pubic nipples. Males with developed external gonads (testes and cauda epididymides) were identified as sexually mature adults. We were also able to distinguish adults from subadults based on the appearance of developed musculus bulbocavernosus at the base of the penis, which is externally visible throughout the year in males that have attained sexual maturity. The reproductive condition of males was classified as either reproductively active (testes and/or cauda epididymides enlarged) or inactive (both gonads regressed) (except juveniles). From 2013 to 2017, we also used digital calipers to measure (to the nearest 0.01 mm) the external size, along the major axis, of the testes and of the cauda epididymides. From 2015 to 2017, after measurements, a total of 31 adult males were collected and brought back to the laboratory for further studies (histological examination, hormone assay and sperm observation, Section 2.5 in detail).

To determine whether little horseshoe bat exhibits delayed fertilization, we collected a total of ten adult females for histological examinations in August (post-lactation and at the beginning of the estrus phase, *n* = 4), December (early-hibernation, *n* = 3) and March (late-hibernation, *n* = 3) of 2017 and 2018. These timings were determined based on our annual life cycle surveys and papers describing the schedule of delayed fertilization in other species (Oxberry, 1979; Kawamoto et al., 1998). Furthermore, during the winters of 2016–2017 and 2017–2018, a total of twenty-three hibernating adult (*n* = 8) and subadult (*n* = 15) females were collected randomly to examine histologically whether they would be fertilizable the following spring.

### 2.4 Female histology

Captured females (total *n* = 33) were carefully transported to the laboratory within 2 h after capture. We euthanized females by intraperitoneal injection of sodium pentobarbital (10 mg/kg) (Morais et al., 2013; Rincón-Vargas et al., 2013). The reproductive tract (ovary, oviduct and uterus) of each individual was extracted and fixed in Bouin’s solution for 24 h. After fixation, the reproductive tract was preserved in 70% ethanol, then dehydrated in graded series in ethanol (70%, 80%, 90%, 95%, and 100%), cleared in xylene and embedded in paraffin. Each sample was sectioned at 4 μm, and stained with hematoxylin–eosin for light microscopy.

For each sampling season, we assessed the presence of developed follicles (antral or Graafian stage: follicles containing an antrum) (Gaisler, 1966; van der Merwe, 1979) in either the left or right ovary, and sperm storage in the uterus. If developed follicles and stored spermatozoa were found during both early- and late-hibernation periods, we presumed that the females had a reproductive cycle representative of delayed fertilization (Oxberry, 1979; Orr and Zuk, 2014). It was also presumed that hibernating females with developed follicles and stored spermatozoa were likely to be fertilized the following spring. Furthermore, we confirmed that females had previously been mated by the presence of a vaginal plug (Gaisler, 1966; Lee, 2020).

### 2.5 Assessing male reproductive physiology

From 2015 to 2017, adult males (total *n* = 31) were collected for histological examination, hormone assay and sperm mobility observation during May and June (hereafter referred to as the “anestrus” period, *n* = 8), during September to early November (hereafter referred to as the “estrus” period, *n* = 10) and during December to March (the hibernation period, *n* = 13). These sampling timings were determined based on seasonal changes in the external size of gonads (testes and cauda epididymides). Although most samples were collected from the Osawa cave, a few males appeared in the cave during spring and early summer, corresponding to the anestrus period. Therefore, to collect males for the experiments, a total of eight bats were captured in the abandoned tunnel (Tokamachi City) during the anestrus period. Males were carefully transported to the laboratory within 2 h of capture. We collected blood samples by cardiac puncture after euthanasia induction by intraperitoneal injection of sodium pentobarbital (10 mg/kg) (Morais et al., 2013; Rincón-Vargas et al., 2013). Blood was centrifuged at 15,000 rpm (18,800 ×g) for 15 min at 4°C. Separated serum was stored at −80°C for later testosterone assay. After the blood collection, we removed testes, cauda epididymides and accessory sex glands (complex of ampullary glands, prostate gland, urethral gland, and vesicular glands) separately. The weight of testis, cauda epididymis and complex of accessory sex glands was weighed. Then, using digital calipers, we measured (to the nearest 0.01 mm along the major axis) the internal size of the testes and the cauda epididymides. The right cauda epididymis was used for histological examination and the left for sperm mobility observation (*n* = 4 bats for each season). For males captured during the anestrus period, we could not collect sufficient volume of blood samples for the hormone assay and sperm for the observation.

Testes and cauda epididymides (total *n* = 12 bats) were fixed in Bouin’s solution for 24 h, and embedded in paraffin after dehydration in a graded series of ethanol (70%, 80%, 90%, 95%, and 100%), then cleared in xylene. They were then sectioned at 4 μm, and stained with hematoxylin–eosin for light microscopy. We examined spermatogenesis in the seminiferous tubules and the presence of spermatozoa in the lumen of the cauda epididymis of each male. To extract spermatozoa for mobility observation, the cauda epididymis was dissected in 1.0 mL of pre-warmed (37°C) HTF medium (Nippon Medical and Chemical Instruments Co., Ltd.). The medium containing spermatozoa was immediately transferred to a Petri dish placed on an inverted phase-contrast microscope with a thermoregulator and CO_2_ incubator. Sperm observations were conducted under 37°C, 5% CO_2_ conditions at ×200 magnification. We randomly chose four spots on the dish, and recorded 5 s videos of each spot to a laptop PC connected with the microscope. At each spot, we counted the number of both motile (swimming) and immotile sperm. A single observer conducted the observation and count of sperm. The testosterone concentration was determined by ELISA Capture/Sandwich using a commercial kit (Testosterone ELISA kit, Item No. 582701, Cayman Chemical, Michigan, United States of America) (Puga et al., 2017). The assay was performed on 12 bats that sufficient serum samples were obtained (anestrus: not applicable, estrus: *n* = 6 out of 10, hibernation: *n* = 6 out of 13). Absorbance was measured at 405 nm on a Multiskan FC (Thermo Scientific, Massachusetts, United States of America) microplate reader. The intra-assay coefficient of variation was 7.7%.

### 2.6 Observation of winter copulation

To determine if copulations occurred during periodic arousals from hibernation, behavioral observations were made at Osawa cave using an infrared night vision camera (Handycam HDR–CX900, Sony Corporation, Tokyo, Japan) from January to early March in 2016 and 2017. The camera was mounted on a tripod with a remotely controllable pan-tilt device, and placed on the cave floor approximately 6 m away from a group of hibernating bats. The camera and pan-tilt device were remotely operated through extension cables using a control-device. Video imagery from the camera was checked on a small portable monitor connected to the control-device. The control-device and monitor were housed in a waterproof box, and placed inside the cave near the entrance in an area not used by bats and accessible during winter without disturbing them. Camera operation was performed by a single observer from the location of the box. All observation devices were powered by a 12V50Ah sealed battery. To confirm that the camera and connected electronics were not producing any noise, we listened directly to the sounds with a bat detector (D240x Ultrasound Detector, Pettersson Elektronik AB, Uppsala, Sweden), which can make ultrasound audible. The devices produced neither audible nor ultrasonic noise, especially in the range of bat echolocation calls (15–110 kHz for bat’s vocalization in Japan, 104 kHz for little horseshoe bats). We also observed the devices using the thermal imaging camera (Thermo Gear G120) in the cave, and confirmed that the devices did not emit any noticeable heat (nor light) sufficient to affect bat behavior.

During the hibernation period, little horseshoe bats tended to periodically arouse from torpor around dusk and were active for several hours (Funakoshi and Uchida, 1980). Thus, for each survey night, observations were started at sunset and continued until no active bats were found. We searched for copulating bats at 30 min intervals by aiming the camera toward the hibernating group and its surroundings. To ascertain copulatory behavior, we referred to characteristics described in the existing literature (Thomas et al., 1979). When copulations were noted, video recordings were made so as to be able to describe the behavior in detail. In January and February 2017, when possible, we captured copulating pairs using a small hand-net soon after coitus, this allowed us to examine whether males had ejaculated successfully. Captured bats were identified to sex, age class, and reproductive condition. For females, we sampled vaginal smears to assess the presence or absence of sperm. Vaginal smears were collected by aspirating distilled water introduced into the vagina with plastic Pasteur pipettes. The collected samples were then placed into 1.5 mL microtubes for each bat. The samples were brought back to the laboratory and stained with Giemsa for light microscopy.

### 2.7 Statistical analysis

To confirm whether external measurements reflect those of internal size or weight of testes and cauda epididymides, correlations between external and internal size, and between external size and weight were analyzed based on Pearson correlation coefficient. For both gonads, external size was significantly correlated with internal size or weight (Supplementary Figure S2; Supplementary Table S1). Thus, we can use external size as a measure of internal condition. The weight of testis, cauda epididymis, and accessory sex glands was compared among seasons (anestrus: *n* = 8, estrus: *n* = 10, hibernation: *n* = 13) using the nonparametric multiple comparison Steel-Dwass test after checking for normality of the data by means of the Shapiro-Wilk test. For statistical comparisons of the testosterone concentration and sperm mobility among seasons, data collected during estrus and hibernation were used. Testosterone concentrations were compared between estrus and hibernation periods using the Wilcoxon signed-rank test (*n* = 6 males for both seasons). To test for differences in sperm mobility between estrus (*n* = 3) and hibernation (*n* = 4) periods, we used the generalized linear model (GLM, family = Poisson). In the GLM, the number of swimming sperm was used as the response variable, and season (estrus and hibernation) was used for the explanatory variable, with the total number of observed sperm as an offset parameter (log-transformed). Furthermore, to compare the composition of female reproductive conditions (determined by histological examination) between bats hibernating solitarily and in groups, we used Fisher’s exact test. All analyses were performed using the statistical software R (R Core Team, 2021, version 4.1.0) run within Rstudio (Rstudio Team, 2021, version 1.4.1717) interface.

## 3 Results

### 3.1 Annual reproductive cycle with female sperm storage

**FIGURE 1:**
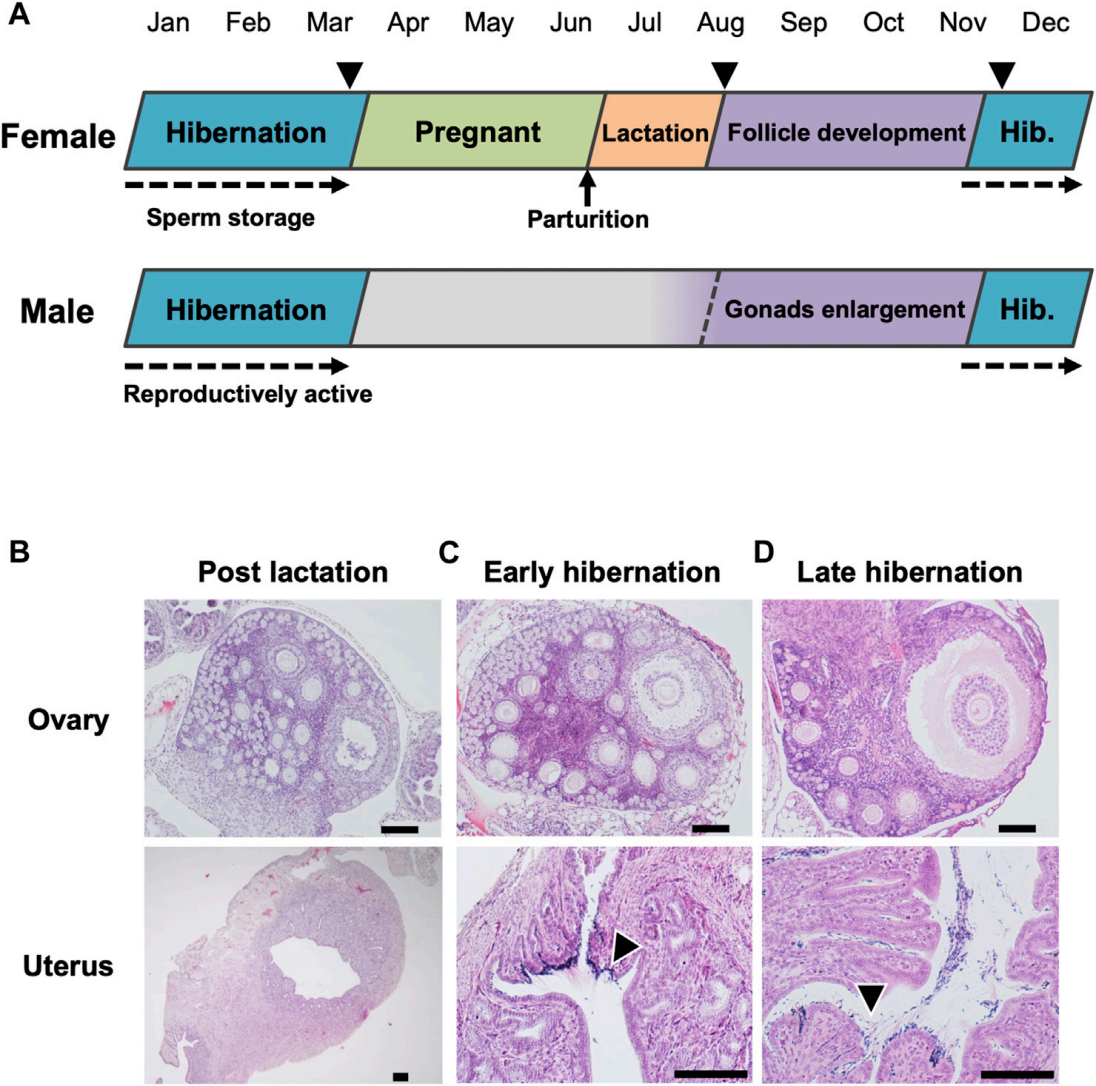
Annual reproductive cycle of the little horseshoe bat (*Rhinolophus cornutus*) in Niigata Prefecture. **(A)** denotes reproductive cycle of both sexes determined by monitoring of reproductive conditions and histological examination of females in **(B–D)**. Arrow heads represent the period of capture for female histological study. The beginning of spermatogenesis was not determined. **(B**–**D)** show ovary and uterus of adult females collected during post-lactation, early hibernation and late hibernation period. Arrow heads in **(C,D)** indicate stored spermatozoa found in the vicinity of the utero-tubal junction. Scale bars on HE stained images are 100 μm.

Pregnancy began after emergence from hibernation, during April to June, and lactating females appeared in the nursery colony with juveniles from July to August (Figure 1A, Supplementary Figure S3A). During 2011–2017, reproductively active males appeared in the cave from autumn through winter (percentage in the captured males: September−November = 64.3%, December−March = 71.2%) (Figure 1A; Supplementary Figure S3B). Histological examination of adult females revealed that post-lactating bats had neither developed ovarian follicles nor spermatozoa, with the exception of one individual with a developing follicle in an ovary (but no spermatozoa) (Figure 1B). During the early- and late-hibernation period, adult females retained developed follicles in their ovaries, and stored spermatozoa in their uteri (Figures 1C, D). Although post-lactating females did not have vaginal plugs, females had plugs during early-hibernation, indicating that follicle development proceeded during autumn, and that copulations had occurred by the beginning of hibernation.

### 3.2 Seasonal changes in male reproductive conditions

**FIGURE 2:**
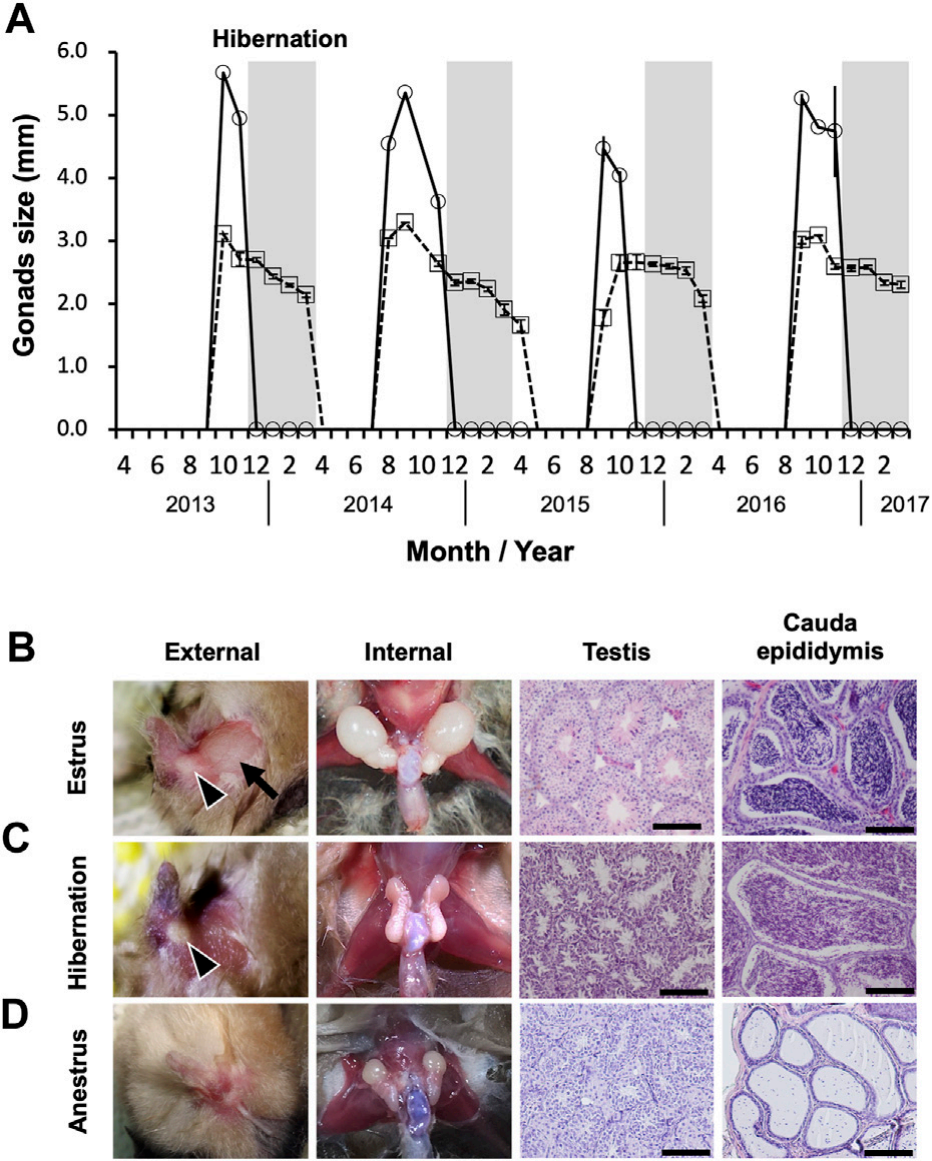
Sperm storage in cauda epididymis during hibernation. **(A)** represents seasonal changes in external testicular (solid line) and cauda epididymis size (dashed line). Measurements are shown as means ± SE. Months without symbols indicate that no males were captured. Light-gray bars represent hibernation. **(B**–**D)** show seasonal changes in external and internal morphology of gonads, and seminiferous tubules of testis and lumen of cauda epididymis. The arrow and arrow heads in **(B,C)** indicate enlarged testis and cauda epididymis, respectively. Scale bars on HE stained images are 100 μm.

**FIGURE 3:**
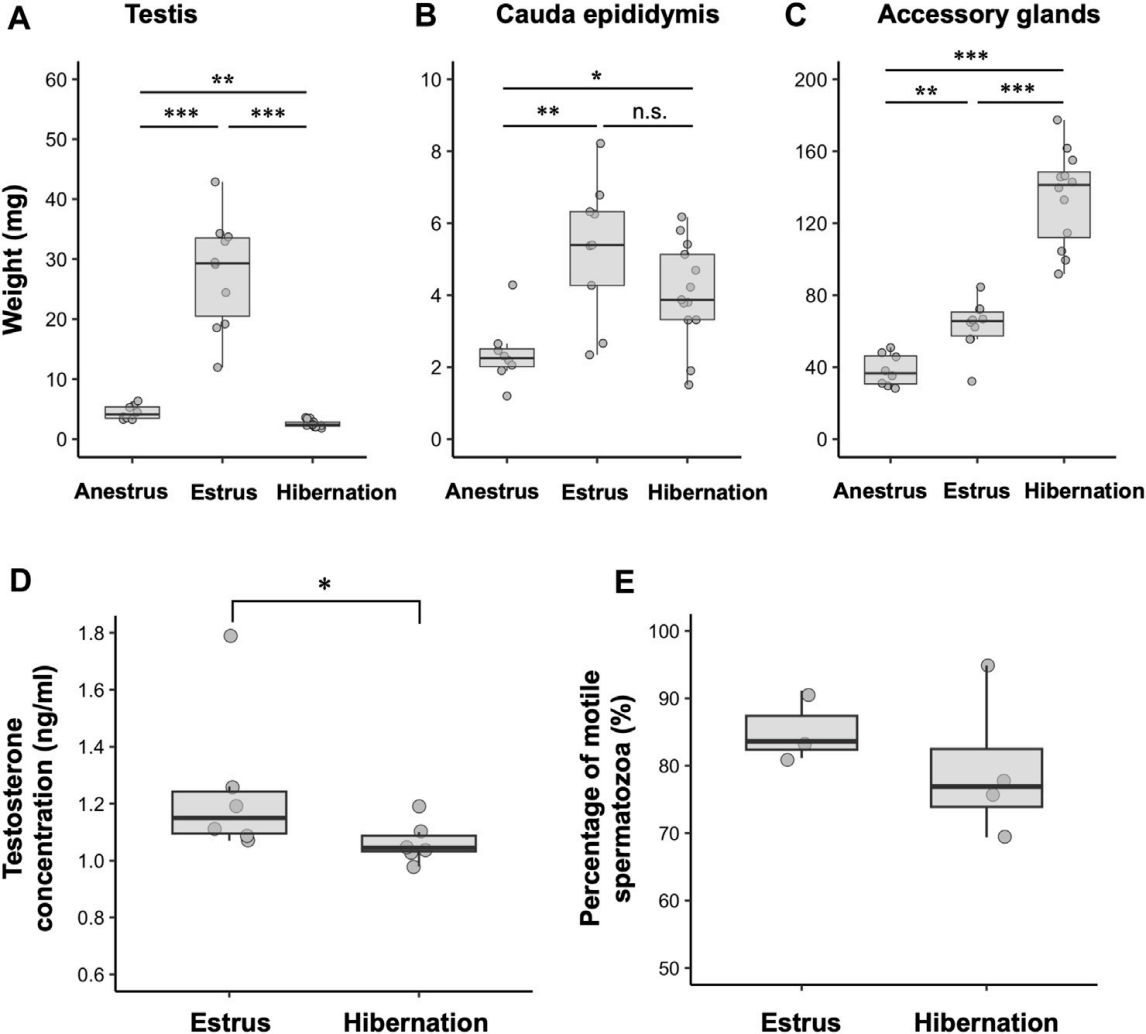
Seasonal changes in male reproductive physiology. **(A–C)** show the weight of testis, cauda epididymis and complex of accessory sex glands during anestrus (*n* = 8), estrus (*n* = 10) and hibernation (*n* = 13). **(D,E)** represent seasonal differences in testosterone concentrations (*n* = 6 for both seasons) and percentages of motile spermatozoa (*n* = 3 in estrus, *n* = 4 in hibernation), respectively. Data are shown using boxplots; the top and bottom of the boxes represent the 75th and 25th percentile, respectively, and lines indicate the median. *: 0.01 < *p* < 0.05, **: 0.001 < *p* < 0.01, ***: *p* < 0.001, n.s.: not significant. Statistics for **(E)** are shown in text and Supplementary Table S2.

Seasonal changes in the external sizes of the testes and cauda epididymides followed similar patterns among years (Figure 2A). Testes enlarged from August to November, peaked in size in September or October (mean ± SE during estrus = 4.74 ± 0.20 mm), then regressed rapidly in December (not measurable externally) (Figure 2A). The cauda epididymides size also reached maximum between August and November (mean ± SE during estrus = 2.78 ± 0.12 mm), and gradually decreased but not completely regressed throughout the hibernation period (mean ± SE during hibernation = 2.37 ± 0.05 mm) (Figure 2A). During estrus, spermatogenesis was confirmed in the seminiferous tubules, and the lumen of the cauda epididymides was filled with spermatozoa (Figure 2B). During hibernation, males stored spermatozoa in the cauda epididymis although the testes atrophied and ceased sperm production (Figure 2C). From spring to early summer (anestrus), we confirmed regressed testes with no sperm production, and scarce spermatozoa in the lumen of the cauda epididymides (Figure 2D). Seasonal differences in the weights of testes and cauda epididymides corresponded with seasonal patterns in their external sizes and histological characteristics. Both testes and cauda epididymides were heaviest during estrus (testes: median = 29.29, range = 11.95–42.88 mg, cauda epididymides: median = 5.82, range = 2.34–10.75 mg) (Figures 3A, B). Testis weight differed significantly among all pairs of seasons, whereas there was no significant difference in the weight of the cauda epididymis between estrus and hibernation (Figure 3A, B; Supplementary Table S2). The accessory sex glands weighed more during hibernation than during estrus and anestrus (anestrus: median = 36.58, range = 28.14–50.89 mg, estrus: median = 65.60, range = 32.06–84.41 mg, hibernation: median = 142.83, range = 99.47–205.7 mg) (Figure 3C; Supplementary Table S2). Testosterone concentration was significantly lower during hibernation than during estrus (estrus: median = 1.15, range = 1.07–1.79 ng/mL, hibernation: median = 1.05, range = 0.98–1.19 ng/mL, Wilcoxon signed-rank test: W = 31, *p* = 0.041) (Figure 3D). There was no significant difference in the proportion of motile spermatozoa between estrus and hibernation (estrus: 85.9%, hibernation: 81.3%, GLM: estimated slope of explanatory variable = −0.054, SE = 0.034, z = −1.566, *p* = 0.117) (Figure 3E; Supplementary Table S3).

### 3.3 Winter forced copulation

**FIGURE 4:**
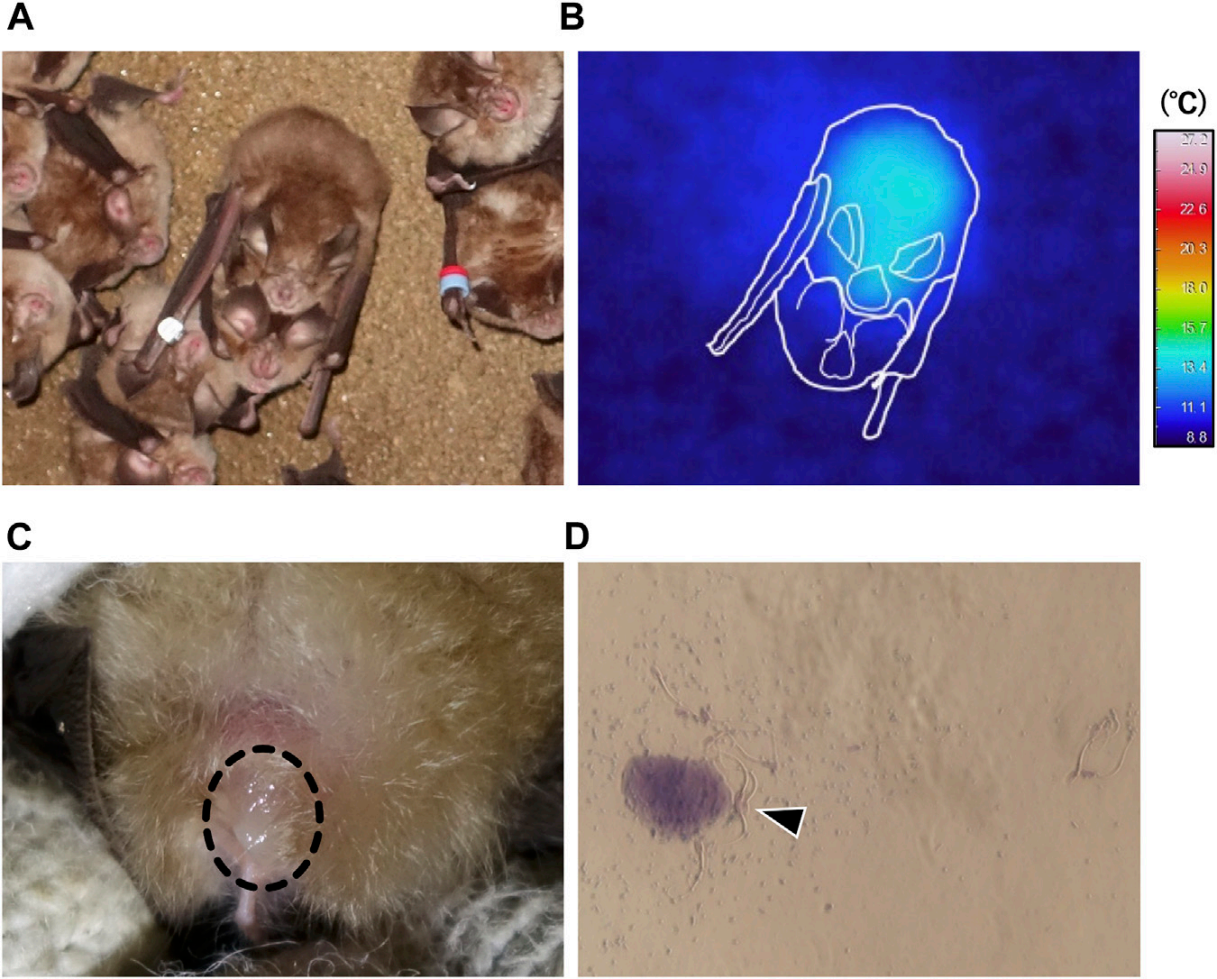
Winter forced copulation by males periodically aroused from torpor. **(A)** is a representative image of a copulating pair. **(B)** shows thermal infrared image corresponding to the pair shown in **(A)**, and approximate outlines (white lines) of bats. **(C)** indicates ejaculated semen (dashed circle) found in the vaginal opening of a post-copulatory female. **(D)** represents spermatozoa (arrow head) collected by vaginal smear.

During hibernation, males arousing from torpor landed beside torpid female bats and attempted to mate with them (Figures 4A, B; Supplementary Video S1), in each case observed copulation was forced. Such events consisted of three behaviors: mounting from the back of the torpid individual, biting the nape of the torpid individual, and attempting intromission (Supplementary Video S1). Recipients often emitted audible vocalizations and struggled. During two survey seasons, amounting to a total of 114 h over 32 days, we observed 70 copulation events of which 29 (41.4%) were successful, including intromission. The remaining 41 events were unsuccessful with males giving up coitus shortly after mounting. We successfully captured seven mating pairs (two of the males escaped) shortly after coitus. All of the males captured after coitus had enlarged cauda epididymides. Five of the seven females were nulliparous in a hibernating group and two were solitary and parous. We confirmed the presence of ejaculated semen on the vaginal opening (Figure 4C) and sperm from smears (Figure 4D; Supplementary Figure S4).

### 3.4 Hibernating females with fertility

**FIGURE 5:**
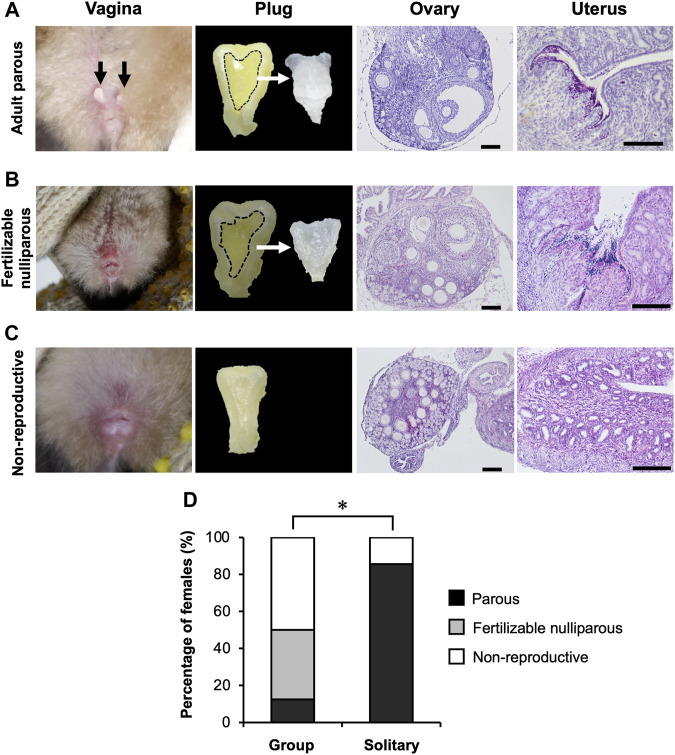
Reproductive conditions of hibernating females. **(A–C)** represent adult parous, fertile nulliparous subadults, and non-reproductive females, respectively. Black arrows in the image of vagina **(A)** indicate pubic nipples (sign of lactating experience). Images of plugs show the cross sections of the vagina after preservation in 10% formalin neutral buffer solution, and the extracted plug. All scale bars on these images are 100 μm. **(D)** shows the comparison in percentages of histologically determined reproductive categories between females hibernating in a group and in those hibernating solitarily (see text for statistics). *: *p* < 0.05 (Fisher’s exact test).

The bats hibernating in the cave formed mixed-sex groups, with groups consistently dominated by adult males (14.1%–69.1% of captured bats) and subadult females (12.9%–56.6% of captured bats) throughout our study period (Supplementary Figure S5; Supplementary Table S4). Twenty-three females (group *n* = 16; solitary *n* = 7) were dissected during two hibernating seasons, and their reproductive condition was determined histologically (Figures 5A–C). The composition of reproductive conditions differed significantly between bats hibernating in a group and those hibernating solitarily (Fisher’s exact test, *p* = 0.0035) (Figure 5D). Few parous females were captured from groups during hibernation (0%–7.7% of captured bats) (Supplementary Figure S5; Supplementary Table S4), and when rarely present they were found solitarily in Osawa cave. All of the parous females that were dissected had spermatozoa in their uteri and developed follicles in their ovaries (Figure 5A). In females captured from hibernating groups, 37.5% of those dissected were externally nulliparous (subadult), but their histological examination showed follicle development, sperm storage and vaginal plug formation (Figure 5B). This indicates that these subadult females had attained fertility in the autumn, and had been copulated with. Fifty percent of females collected from hibernating groups showed no signs of insemination or fertility (Figure 5C).

## 4 Discussion

### 4.1 Male adaptations for sperm competition

We found that male little horseshoe bats stored spermatozoa in their cauda epididymides, and attempted to copulate despite having regressed testes during the hibernation period. This indicates that epididymal sperm storage during winter allows for extended opportunities for mating. Females hibernating in groups were mainly nulliparous (based on external examination), but some of them were ready for fertilization. For males, fertile females in the hibernaculum appear to be available as potential mates. Females stored spermatozoa during hibernation and gave birth to just one offspring per year, indicating that males experience reduced certainty of successful fertilization. It has been reported that the probability of sperm remaining in the female reproductive tract declines over time as females expels sperm from previous copulations (Krutzsch, 1975; Tidemann, 1993). Therefore, forced copulation during winter may provide additional opportunities for successful fertilization by allowing males to pass more sperm to their mates, and to displace the ejaculates of previous rivals (Edward et al., 2015). Winter copulations may also help to compensate for missed mating opportunities before hibernation (Boyles et al., 2020). Although the number of cases described is still limited, male sperm storage and winter copulations have also been observed in several bat species that have delayed fertilization (Thomas et al., 1979; Boyles et al., 2006). These male characters may reflect adaptations for sperm competition, which is facilitated by delayed fertilization (Orr and Zuk, 2014; Edward et al., 2015). In this study, we were unable to assess the effectiveness of winter copulations in terms of fertilization success; however, the adaptive significance of this behavior seems worthy of further investigation. Future research, clarifying whether winter copulations including physiological mechanisms enabling males to express sexual behavior have evolved widely among species with delayed fertilization, is also needed.

Two interpretations of the characteristics of males attempting winter copulations are possible. The first interpretation is that a difference in competitiveness among males may be associated with winter copulations. Previous research has shown that competitively dominant males can monopolize females for mating during late summer (Racey and Entwistle, 2000; Senior et al., 2005). Paternity success is also biased towards just a few males in a breeding colony, and success if often determined by age (increased with age) (Watt and Fenton, 1995; Heckel and von Helversen, 2002; Rossiter et al., 2006). If dominant males can out-compete younger and/or inferior males early in the mating season, excluded males might keep attempting to mate during winter. The second interpretation is that it is possible that males in better condition can afford to copulate during winter. For hibernating bats, it is important that they optimize their energy expenditure for successful overwintering (Willis, 2017; Boyles et al., 2020). Arousal from torpor can account for up to 75% of overwintering energy expenditure (Thomas et al., 1990). Euthermic activities following arousal are energetically expensive and lead to fat reserve depletion (Boyles and Willis, 2010). Foraging opportunities outside roosts are drastically reduced during harsh weather conditions and periods of low prey availability. Even though bats are able to forage when periodically arousing from torpor, it may be insufficient for them to fuel their energy reserves, except at low latitudes, in warmer wintering regions (Boyles et al., 2020). Thus, males storing sperm are likely to face a trade-off during hibernation between investing in survival or in mating behavior (energy saving vs. increasing mating opportunities). For example, in the little brown bat (*Myotis lucifugus*), males arouse longer than females in winter, and individuals in better condition exhibit longer periods of arousal (Czenze et al., 2017). Hibernating males with larger fat reserves may be able to invest more energy in copulatory behavior during periodic arousal from torpor.

Females collected during hibernation had copulatory plugs in their vaginas. Such plugs are formed after mating from substances produced by either sex (Devine, 1977; Voss, 1979). In bats with delayed fertilization, copulatory plugs are typically interpreted as a form of “mate guarding” by males to prevent subsequent copulations (Orr and Zuk, 2014). Because male little horseshoe bats can continue to copulate during the period of sperm storage, their previous mates are likely to be re-mated by other males. Thus, mate guarding by means of plugs may be a necessary way of defending their mates and out-competing other males by reducing the mating chances of their rivals (Edward et al., 2015). Additionally, copulatory plugs also play a role in facilitating sperm transport within the reproductive tract by preventing the female from sperm dumping, as, for example, in mice (*Mus musculus*) (Dean, 2013). Copulatory plugs may be beneficial in situations in which early mating can secure advantageous storing positions for the spermatozoa in the vicinity of the oviduct where fertilization occurs (Racey, 1979). However, female bats have often been observed to remove plugs and dump sperm from a previous copulation (Pearson et al., 1952; Phillips and Inwards, 1985), and male bats also have penile weapons, such as spines, which may be used to remove plugs (Armstrong, 2005). It is difficult, therefore, to conclude whether copulatory plugs can completely prevent rival males from later copulations, thus further investigation to facilitate an understanding of the function of such plugs during hibernation is needed.

For male bats, winter forced copulations appear to be advantageous, allowing them to obtain mates while avoiding pre-copulatory female choice. For female bats, it is not possible to determine costs/benefits of forced copulations during hibernation in this study. However, it has been recognized that male sexual coercion could have negative effects on female fitness (Clutton-Brock and Parker, 1995; Cassini, 2020). For example, in mammals, forced copulations increase morbidity and mortality risk of females, and reduce female reproductive success (Smuts and Smuts, 1993; Reale et al., 1996; Linklater et al., 1999; Manguette et al., 2019). These effects are presumed to be caused by increased physical injury or disease transmission during repeated copulations, and by fertilization with sperm of lower-quality males (Edward et al., 2015). To reduce these costs, females often develop counterstrategies (Cassini, 2020). Sexual segregation and avoiding male’s territory are interpreted as female responses to sexual coercion by males (Cassini, 2020). In our study site, although subadult females hibernated in groups with adult males, adult females were rarely present in the same hibernaculum. Furthermore, females hibernating in groups often experienced forced copulation. Females having experienced forced copulations as subadults may avoid winter roost sites with adult males. If sexual coercion also detrimentally affects female little horseshoe bats, choosing different hibernacula may develop as a counterstrategy to avoid disturbance by males. However, it should be noted that roost site segregation is also determined by differences in thermoregulatory, microclimatic and energy requirements between sexes (Senior et al., 2005; Angell et al., 2013; Stawski et al., 2014). During hibernation, adult females tend to be more conservative with their energy reserves than either adult males or younger bats (Jonasson and Willis, 2011), because emerging from hibernation in spring in good body condition is important for initiating gestation without delay (Willis, 2017). Thus, adult females may choose their wintering roosts based on the availability of suitable environmental conditions for conserving energy reserves. This might also explain why adult females are scarce at our study site.

### 4.2 Physiological mechanisms related to winter copulation

Testicular development with spermatogenic activity ceases as winter commences, whereas cauda epididymides and accessory sex glands are retained during hibernation. Testicular production of gametes is sensitive to cooling temperatures (Komar et al., 2022). Even among heterothermic species, cell growth and steroidogenesis are greatly reduced at lower body temperatures (<20°C), and enzymatic reactions in the testes work efficiently only within limited temperatures (Davis et al., 1963; LeVier and Spaziani, 1968). Multiday torpor during winter is likely to suppress testicular activity in the little horseshoe bat. Conversely, low body temperature during hibernation may be favorable for long-term epididymal sperm storage (Geiser and Brigham, 2012). At low temperatures, the metabolic rate of spermatozoa is reduced, and this inhibits the production of harmful metabolites (e.g., reactive oxygen species), which can damage sperm cells (Gibb and Aitken, 2016; Dziekońska et al., 2020). In bats, the fluid in the epididymal lumen during hibernation shows high levels of osmolarity leading to respiratory reduction, energy conservation, and motility preservation of stored spermatozoa (Crichton et al., 1993; Crichton et al., 1994). Furthermore, it has been suggested that the hyperosmolar environment in the lumen is associated with formation of a blood-epididymal barrier (close relations among epididymal epithelial cells), particularly during the period of sperm storage (Ahiezer et al., 2015). Hibernation-associated epididymal environments may provide suitable conditions for long-term storage and maintenance of viable spermatozoa.

The accessory sex glands of little horseshoe bat weighed most during hibernation. The activities of these glands play an important role in seminal fluid secretion and ejaculation (Clement and Giuliano, 2016). Generally, protein synthesis is suppressed by hibernation (Frerichs et al., 1998; Drew et al., 2007), so in winter glands are likely to be filled with seminal fluid maintained in storage rather than with newly synthesized fluid. In addition, seminal fluid may have remained in the glands simply because of the decreased frequency of copulations during winter compared to the autumn mating season. Although detailed mechanisms have not been described, the innervation control relating to contractions of the glands may be involved in keeping seminal fluid in accessory sex glands throughout the hibernation period.

Interestingly, male bats engaged in sexual behavior even during testicular regression and while testosterone levels were lowered during hibernation. This suggests that higher levels of gonadal steroid secretion may not be necessary for activation and maintenance of sexual behavior during winter. Our results are in accordance with previous findings obtained from gonadectomized bats. After castration big brown bat (*Eptesicus fuscus*), males continued sexual behavior despite reduced androgen levels (Mendonça et al., 1996). Although possible mechanisms underlying this phenomenon are still unclear in bats, there could be an alternative system for expressing sexual behavior in the regressed gonads. Among non-hibernating animals, such as mice, birds, and nonhuman primates, sexual behaviors are also not completely abolished in the absence of testicular activity (Phoenix et al., 1973; Pradhan et al., 2010; David et al., 2022). One possible explanation for gonadal steroid-independent sexual behavior is that social interactions can induce temporally acute androgen increases (<1 h) (Marler and Trainor, 2020). Importantly, such a transient surge could be of extragonadal origin (Wingfield et al., 2001; Balthazart and Ball, 2006). Detailed mechanisms for the transient synthesis of androgen have been described in seasonal breeding birds. In song sparrows (*Melospiza melodia*), aggressive encounters among males rapidly increase local androgen synthesis in specific regions of the brain during the non-breeding season, when gonads have regressed (Pradhan et al., 2010). This finding also highlights that seasonal breeders can shift from systemic to local sex steroid signaling in the absence of gonadal activity (Pradhan et al., 2010). Local synthesis of steroids appears to be a reasonable mechanism to avoid the energetic cost of maintaining high systemic hormone levels during the non-breeding season (Wingfield et al., 2001). The same may be true for hibernating mammals as these also face seasonal energetic constraints. It has not been shown whether hibernating bats have similar mechanisms, but their extragonadal local steroid synthesis may help to explain why male bats can retain sexual behavior even under the constraints of hibernation.

## Data Availability

The original contributions presented in the study are included in the article/Supplementary Material, further inquiries can be directed to the corresponding author.
