# Supplementary material for: Mating in the cold. Prolonged sperm storage provides opportunities for forced copulation by male bats during winter
Source: Front Physiol. 2023 Sep 6;14:1241470. doi: 10.3389/fphys.2023.1241470 (PMC10511888; doi:10.3389/fphys.2023.1241470)
Supplement: Supplementary file 3 [file DataSheet1.DOCX]

Supplementary Material

Mating in the Cold. Prolonged sperm storage provides opportunities for forced copulation by male bats during winter.

Takahiro Sato*, Toshie Sugiyama, Tsuneo Sekijima

*** Correspondence:** Takahiro Sato: satoj@tokushima-u.ac.jp

# Supplementary Data

Data sheets used in this study will be uploaded separately as Excel files.

# Supplementary Figures and Tables

## Supplementary Figures


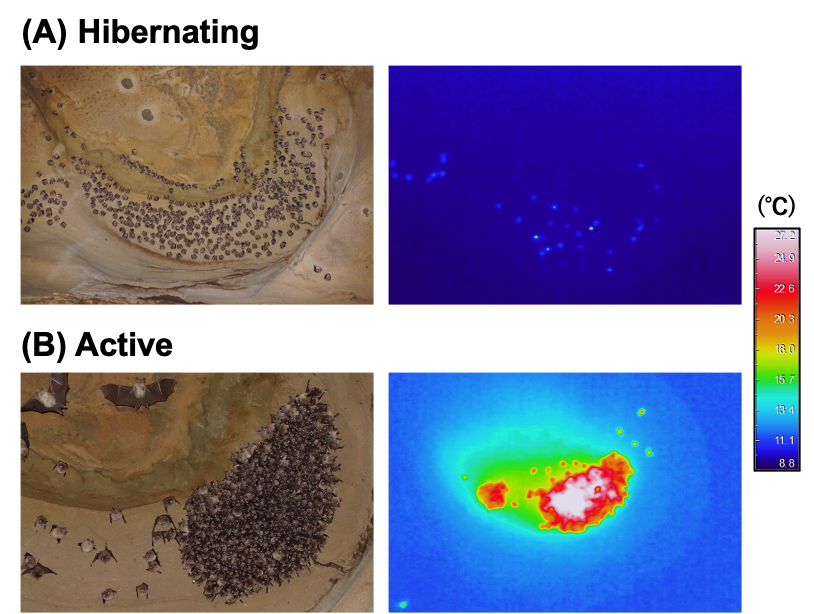


**Supplementary Figure 1.** Roosting bats in Osawa cave. (A) shows a hibernating group on the cave ceiling (left panel), and a representative thermal infrared image of torpid bats (right panel). (B) represents a maternity bat group in early summer (left panel) and a thermal infrared image (right panel) corresponding to the group shown in the left panel. Bats entering torpor showed no noticeable movements, whereas euthermic individuals show apparent movements of their head (emitting echolocation calls).


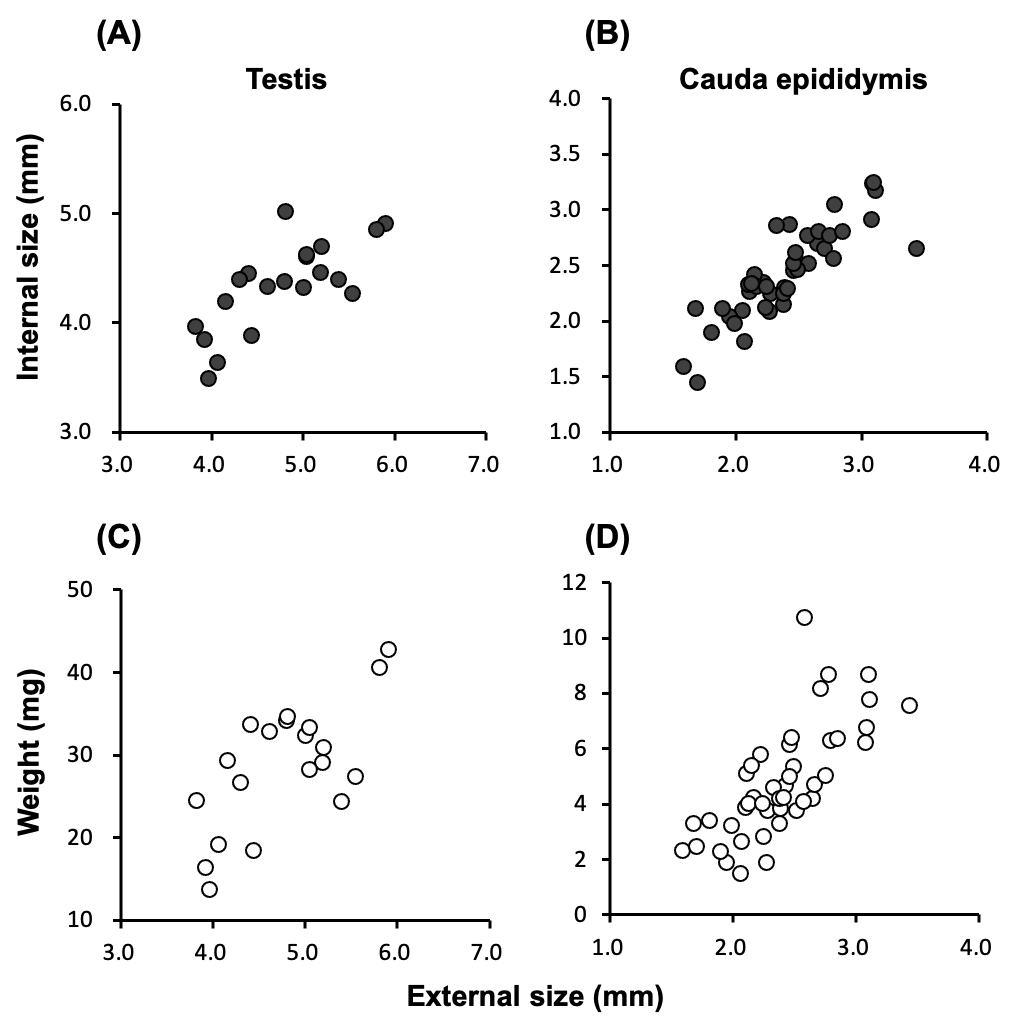


**Supplementary Figure 2.** (A) and (B) represent relationships between external and internal size of testes and cauda epididymides, respectively. (C) and (D) show relationships between external size and weight of testes and cauda epididymides, respectively. All pairs of correlations are statistically significant (see Supplementary Table 1 for statistics).


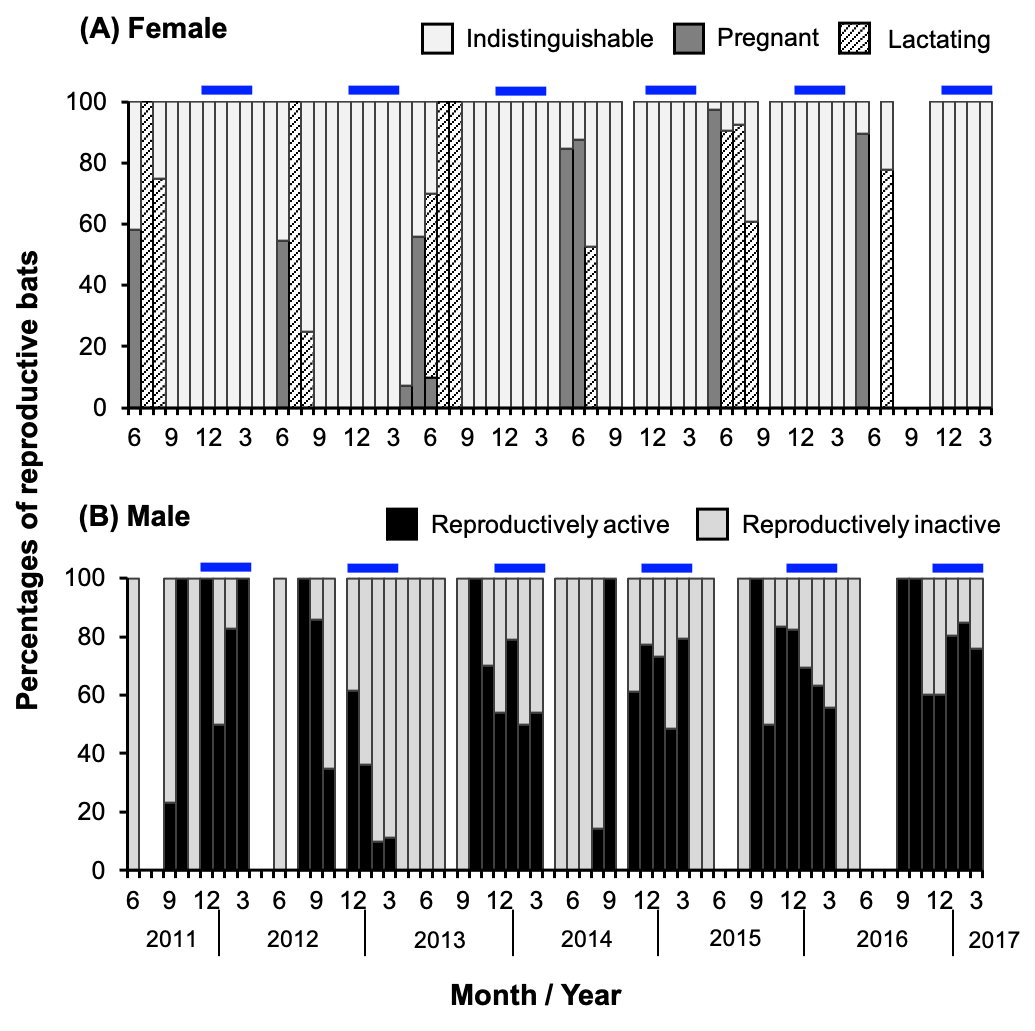


**Supplementary Figure 3.** Seasonal changes in reproductive conditions of bats. Percentages of reproductive bats (excluding juveniles) among captured individuals are shown for each month (the percentage is calculated for each sex). Blue bars on graphs indicate hibernation. In June 2016, we did not conduct capture procedure to avoid excessive disturbance to maternity colony.


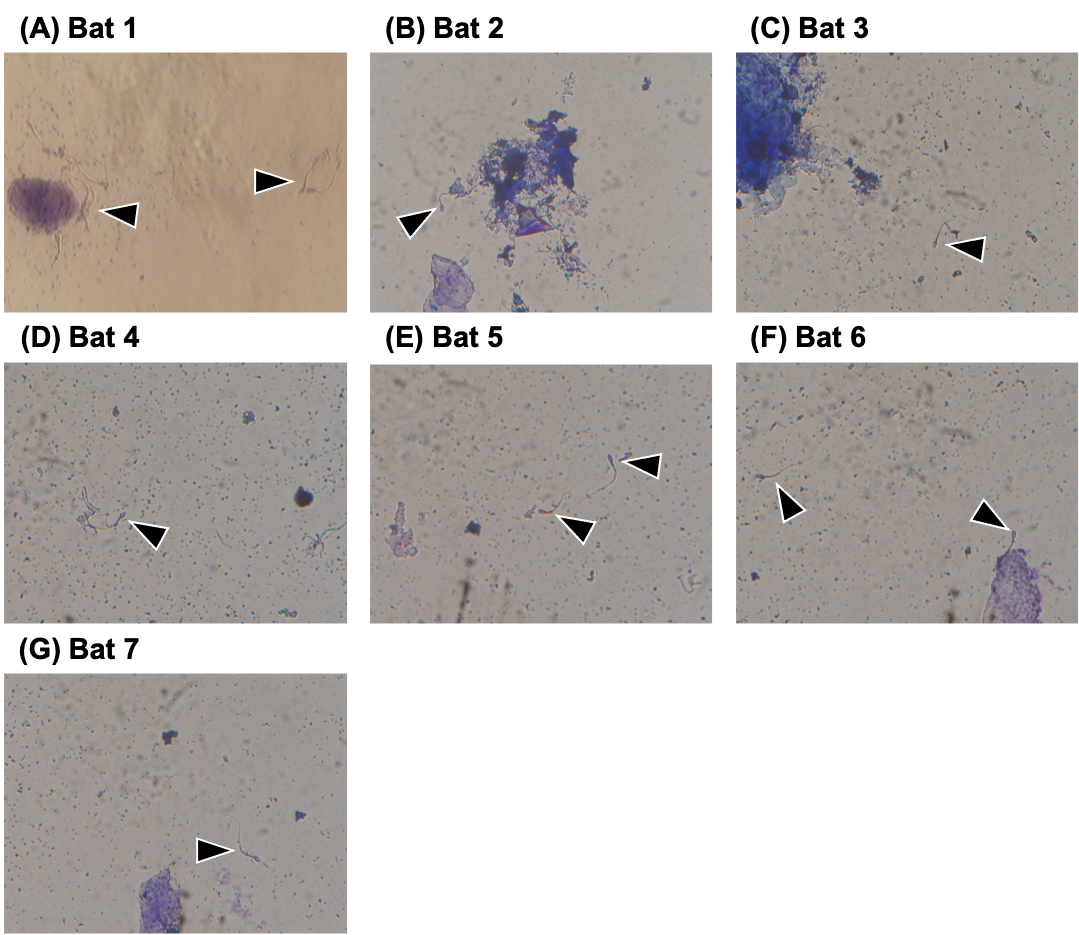


**Supplementary Figure 4.** Vaginal smears of copulatory pairs. Arrow heads in each image indicate spermatozoa.


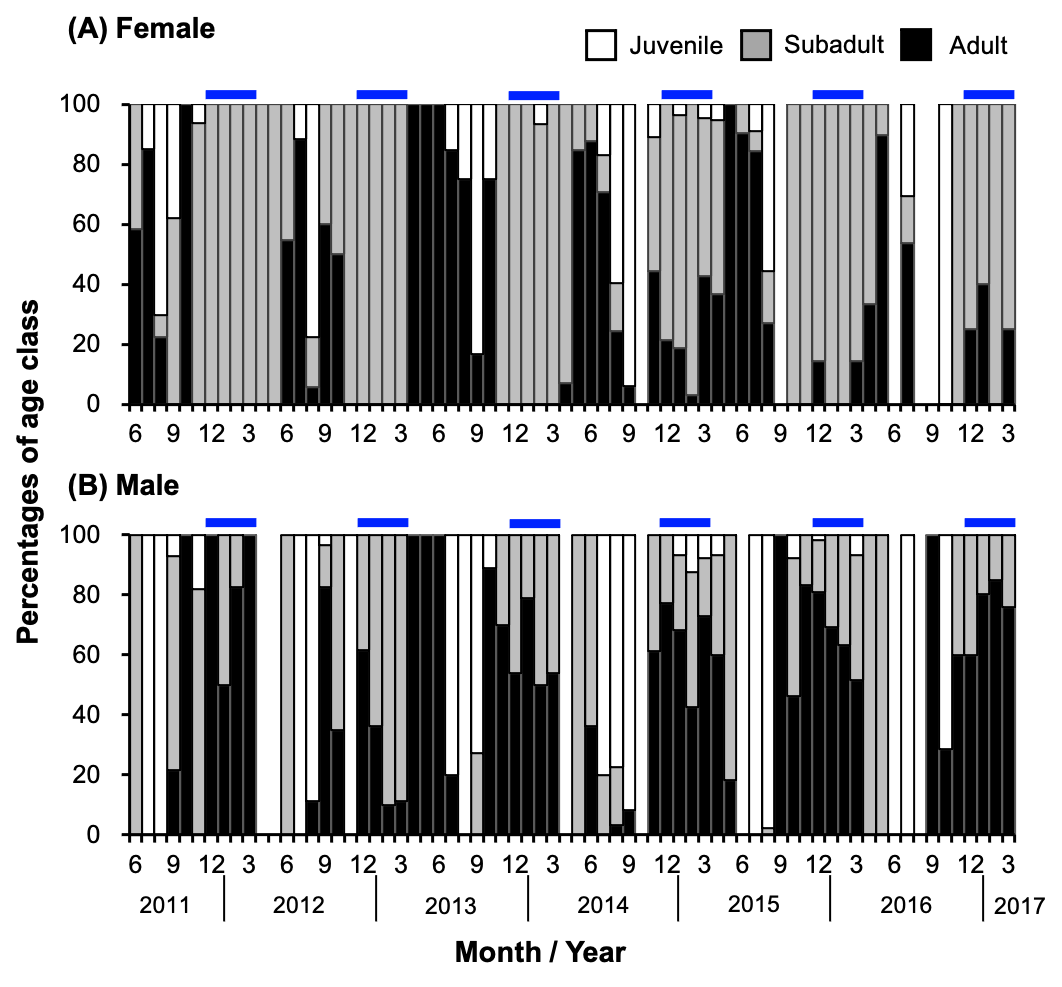


**Supplementary Figure 5.** Seasonal changes in age class compositions. Percentages of age classes among captured bats are shown for each month (the percentage is calculated for each sex). Blue bars on graphs indicate hibernation. In June 2016, we did not conduct capture procedure to avoid excessive disturbance to maternity colony.

**2.2 Supplementary Tables**

**Supplementary Table 1.** Relationships between external and internal size, and between external size and weight of gonads based on Pearson's product-moment correlation.

| Gonads* | Pair | t | df | p-value | Coefficient |
| --- | --- | --- | --- | --- | --- |
| Testis | External vs. Internal | 4.76 | 18 | 0.00016 | 0.747 |
|  | External vs. Weight | 4.03 | 18 | 0.00079 | 0.688 |
| Cauda epididymis | External vs. Internal | 10.75 | 42 | 1.26E-13 | 0.856 |
|  | External vs. Weight | 7.00 | 42 | 1.43E-08 | 0.734 |

*, n = 10 males for testis, n = 22 males for cauda epididymis. Both sides (left and right) of gonads were used in analyses separately.

**Supplementary Table 2.** Results of Steel-Dwass multiple comparison test on the mean weight of each reproductive organ (testis, cauda epididymis and accessory glands).

| Organs | Comparison | W | p-value |
| --- | --- | --- | --- |
| Testis | Anestrus-estrus | 5.026 | 0.0004 |
|  | Estrus-hibernation | -5.701 | < 0.00001 |
|  | Hibernation-anestrus | -4.609 | 0.0015 |
| Cauda epididymis | Anestrus-estrus | 4.398 | 0.0023 |
|  | Estrus-hibernation | -2.807 | 0.1171 |
|  | Hibernation-anestrus | 3.330 | 0.0482 |
| Accessory glands | Anestrus-estrus | 4.398 | 0.0024 |
|  | Estrus-hibernation | 5.701 | < 0.00001 |
|  | Hibernation-anestrus | 5.326 | < 0.00001 |

**Supplementary Table 3.** Result of generalized linear model on the number of motile spermatozoa between seasons.

| Variable | Estimate | Std. Error | z value | Pr (>\| z \|) |
| --- | --- | --- | --- | --- |
| Intercept | -0.15263 | 0.02666 | -5.725 | 1.03E-08 |
| Season (hibernation) | -0.05389 | 0.03442 | -1.566 | 0.117 |

**Supplementary Table 4.** Age class composition of each sex during hibernation.

| Year | Sex | Age class | Captured | % of each sex | % of total |
| --- | --- | --- | --- | --- | --- |
| 2011−2012 | Female | Adult | 0 | 0.0 | 0.0 |
|  |  | Subadult | 43 | 100.0 | 24.6 |
|  |  | Juvenile | 0 | 0.0 | 0.0 |
|  |  | Total | 43 |  |  |
|  | Male | Adult | 121 | 91.7 | 69.1 |
|  |  | Subadult | 11 | 8.3 | 6.3 |
|  |  | Juvenile | 0 | 0.0 | 0.0 |
|  |  | Total | 132 |  |  |
| 2012−2013 | Female | Adult | 0 | 0.0 | 0.0 |
|  |  | Subadult | 56 | 100.0 | 56.6 |
|  |  | Juvenile | 0 | 0.0 | 0.0 |
|  |  | Total | 56 |  |  |
|  | Male | Adult | 14 | 32.6 | 14.1 |
|  |  | Subadult | 29 | 67.4 | 29.3 |
|  |  | Juvenile | 0 | 0.0 | 0.0 |
|  |  | Total | 43 |  |  |
| 2013−2014 | Female | Adult | 0 | 0.0 | 0.0 |
|  |  | Subadult | 43 | 97.7 | 31.4 |
|  |  | Juvenile | 1 | 2.3 | 0.7 |
|  |  | Total | 44 |  |  |
|  | Male | Adult | 54 | 58.1 | 39.4 |
|  |  | Subadult | 39 | 41.9 | 28.5 |
|  |  | Juvenile | 0 | 0.0 | 0.0 |
|  |  | Total | 93 |  |  |
| 2014−2015 | Female | Adult | 18 | 19.1 | 7.7 |
|  |  | Subadult | 74 | 78.7 | 31.5 |
|  |  | Juvenile | 2 | 2.1 | 0.9 |
|  |  | Total | 94 |  |  |
|  | Male | Adult | 90 | 63.8 | 38.3 |
|  |  | Subadult | 41 | 29.1 | 17.4 |
|  |  | Juvenile | 10 | 7.1 | 4.3 |
|  |  | Total | 141 |  |  |
| 2015−2016 | Female | Adult | 5 | 6.9 | 2.0 |
|  |  | Subadult | 67 | 93.1 | 26.9 |
|  |  | Juvenile | 0 | 0.0 | 0.0 |
|  |  | Total | 72 |  |  |
|  | Male | Adult | 122 | 68.9 | 49.0 |
|  |  | Subadult | 52 | 29.4 | 20.9 |
|  |  | Juvenile | 3 | 1.7 | 1.2 |
|  |  | Total | 177 |  |  |
| 2016−2017 | Female | Adult | 10 | 31.3 | 5.8 |
|  |  | Subadult | 22 | 68.8 | 12.9 |
|  |  | Juvenile | 0 | 0.0 | 0.0 |
|  |  | Total | 32 |  |  |
|  | Male | Adult | 106 | 76.3 | 62.0 |
|  |  | Subadult | 33 | 23.7 | 19.3 |
|  |  | Juvenile | 0 | 0.0 | 0.0 |
|  |  | Total | 139 |  |  |

**3 Supplementary Video**

Supplementary video will be uploaded separately as mp4 file.

**Supplementary Video 1.** Copulatory behavior of bats observed during hibernation. The first scene shows a male (identified by the ring attached on his forearm) arousing from torpor and walking around hibernating bats. In the second scene, another individual can be seen mounting torpid bats, thrusting its penis repeatedly (third scene) and intromission (fourth scene). The third and fourth scenes are of the same individual.
